# Severe Hemolytic Disease of the Fetus Treated with Serial Intrauterine Transfusions: A Case Report and Review of Current Management

**DOI:** 10.3390/life15121875

**Published:** 2025-12-08

**Authors:** Olga Olejniczak, Jakub Kornacki, Ewa Wender-Ożegowska

**Affiliations:** 1 Department of Reproduction, Chair of Reproduction and Perinatal Medicine, Poznan University of Medical Sciences, 61-701 Poznan, Poland; 2 Doctoral School, Poznan University of Medical Sciences, 60-701 Poznan, Poland

**Keywords:** intrauterine transfusion, hemolytic disease of the fetus and newborn, rhesus incompatibility, fetal hydrops

## Abstract

Hemolytic disease of the fetus and newborn (HDFN) is a severe complication of pregnancy caused by maternal alloimmunization to fetal red blood cells, leading to significant perinatal morbidity and mortality. The prognosis is particularly poor in cases complicated by fetal hydrops. Prophylactic administration of anti-D immunoglobulin—during pregnancy, postpartum, and after events causing fetomaternal hemorrhage—has substantially reduced the incidence and severity of Rh-related HDFN. Nevertheless, the condition can still occur, either due to omitted prophylaxis or undetected fetomaternal hemorrhage. Definitive management often requires invasive interventions, including cordocentesis and intrauterine transfusions (IUTs), sometimes repeated multiple times, while the optimal timing of delivery remains uncertain, necessitating a careful balance between prematurity and ongoing fetal risk. We report the case of a 35-year-old multipara whose two most recent pregnancies were complicated by HDFN. The first affected pregnancy had a mild course, whereas the second was severe, necessitating multiple intrauterine transfusions (IUTs) throughout gestation. Despite an extremely low initial fetal hematocrit (4.5%), severe hydrops, and the requirement of six intrauterine transfusions (IUTs) during the pregnancy, the infant was delivered at 36 weeks’ gestation with a favorable postnatal outcome. This case report provides a comprehensive overview of intrauterine transfusion methodology, post-transfusion pregnancy monitoring, timing of successive IUTs, and optimal delivery planning in pregnancies complicated by HDFN.

## 1. Introduction

Hemolytic disease of the fetus and newborn (HDFN) is a potentially life-threatening condition arising when an RhD-negative mother carries an RhD-positive fetus, typically due to paternal inheritance of the RhD antigen [1,2].

Maternal sensitization arises when fetal RhD-positive red blood cells enter the maternal circulation, typically during delivery or invasive procedures, prompting an immune response that produces antibodies causing hemolysis of fetal erythrocytes [3].

HDFN most often affects subsequent pregnancies, as initial maternal alloimmunization produces IgM antibodies, which do not cross the placenta. IgG antibodies, responsible for fetal erythrocyte destruction, are generated later, generally after the sensitizing pregnancy [4]. Thus, clinical effects of sensitization typically appear in subsequent pregnancies, when maternal IgG anti-D antibodies cross the placenta and target fetal RhD-positive erythrocytes, markedly increasing the risk of HDFN [5,6,7].

Heightened awareness and prophylaxis—administration of anti-D immunoglobulin at 28–30 weeks, postpartum dosing based on delivery type, and additional doses after events causing fetomaternal hemorrhage—have markedly reduced neonatal mortality from Rh incompatibility [8,9,10].

Despite preventive advances, HDFN remains a concern, particularly in low-resource settings lacking access to anti-D immunoglobulin, and can still occur in high-income countries due to missed prophylaxis or unrecognized fetomaternal hemorrhage [6,11].

Advances in ultrasonography have enabled noninvasive monitoring of alloimmunized pregnancies, primarily through Doppler assessment of peak systolic velocity in the fetal middle cerebral artery. Notably, some data suggest that MCA-PSV may not accurately reflect fetal anemia after repeated procedures [12,13,14,15].

Once diagnosed, HDFN is primarily managed with invasive procedures such as cordocentesis and intrauterine transfusion, often requiring multiple interventions [16]. The optimal timing of delivery remains unclear, as clinicians must balance the risks of prematurity against the potential need for additional invasive interventions.

Optimal strategies for surveillance and management of pregnancies complicated by HDFN remain the subject of ongoing research, as the condition continues to be associated with substantial risks of fetal morbidity and mortality, even when identified promptly and managed appropriately.

This study describes a case of severe HDFN managed through serial IUTs. The fetus presented with a critically low hematocrit and significant hydrops, suggesting an extremely poor prognosis; however, intervention ultimately led to a favorable outcome. Building on this clinical course, we outline potential causes of maternal alloimmunization and preventive measures, approaches to fetal assessment, and principles guiding the timing of repeat procedures and delivery.

## 2. Materials and Methods

Each intrauterine transfusion (IUT) was performed under aseptic conditions using a 20-gauge needle. A prophylactic dose of cefazolin was administered 30 min prior to the procedure. To reduce fetal movements, 10 mg of diazepam was given intramuscularly to the mother a few minutes before the procedure. Additionally, an infusion of the oxytocin receptor antagonist atosiban was initiated 30 min before each IUT and continued for 8 h post-procedure to prevent uterine contractions induced by needle insertion.

Although puncture at the placental cord insertion site was preferred, the ‘free-loop’ needling technique was used in all cases due to unfavorable anatomical conditions, such as a dense vascular network along the anticipated needle path. Immediately following fetal blood sampling for a complete blood count, rocuronium was administered to induce fetal paralysis prior to transfusion. Subsequently, without withdrawing the needle from the vessel, the calculated volume of irradiated O-negative red blood cell concentrate was transfused. The transfusion volume was determined using the calculator available on the Fetal Medicine Barcelona website: https://fetalmedicinebarcelona.org/calc (accessed on 1 December 2024).

## 3. Case Presentation

### 3.1. Description of the Case

#### 3.1.1. Medical History and Symptoms

A 35-year-old gravida 3, para 2 woman was referred to a tertiary center at 37 weeks’ gestation for suspected fetal anemia, prompted by the presence of maternal red-cell antibodies and elevated middle cerebral artery peak systolic velocity (MCA-PSV). Doppler confirmed anemia (MCA-PSV 89.25 cm/s), with associated hepatosplenomegaly, cardiomegaly, and tricuspid regurgitation.

Maternal serology demonstrated anti-RhD antibodies with a titer of 1:512. Anti-RhC antibodies were also present, though detectable only on enzymatic testing and therefore not quantifiable. These serologic and ultrasound findings confirmed the diagnosis of HDFN.

Labor was initiated at 37 weeks because of suspected fetal compromise, but an emergency cesarean section was required due to fetal distress. Postpartum anti-RhD immunoglobulin was not indicated in the setting of established alloimmunization.

No detailed information was available regarding the patient’s previous pregnancies. It remains unclear whether anti-D prophylaxis had been omitted or whether any episodes of fetomaternal hemorrhage had occurred. According to the patient’s account, anti-D prophylaxis had been administered; however, this cannot be confirmed due to the unavailability of medical records from her prior pregnancies.

**Figure 1:**
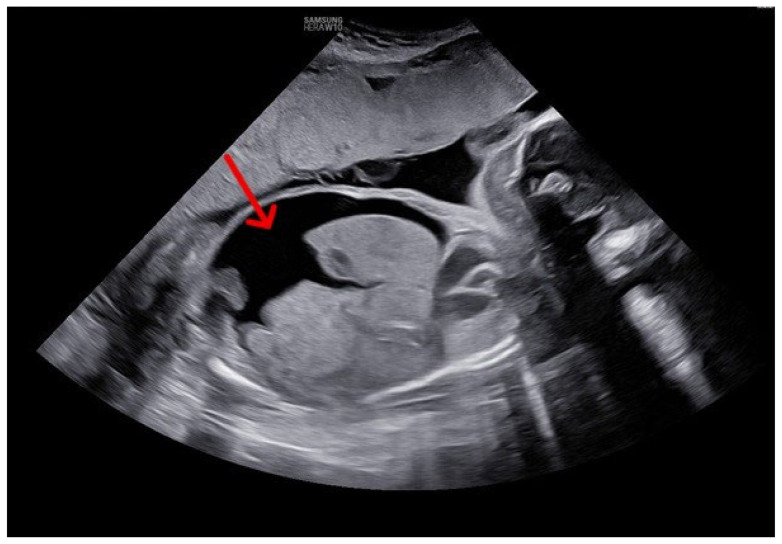
Ultrasound image obtained at the time of hospital admission and diagnosis, demonstrating massive fetal ascites (red arrow) at 24 weeks of gestation. Sagittal view of the fetal abdomen.

One year later, in her fourth pregnancy (gravida 4, para 3), the patient was admitted at 23 weeks after ultrasound screening identified fetal hydrops and maternal alloantibodies. Doppler assessment showed an MCA-PSV of 65.31 cm/s (2.14 MoM), indicating moderate to severe fetal anemia. Additional findings included tricuspid regurgitation, generalized subcutaneous edema, and massive ascites (Figure 1).

Maternal serology confirmed both IgM and IgG anti-RhD alloantibodies, with an anti-RhD titer of 1:1024. Anti-RhC antibodies were also present but could not be quantified due to low serum reactivity. The RhD-positive paternal phenotype again placed the fetus at elevated risk for severe HDFN.

#### 3.1.2. Treatment and Intervention

An IUT was planned for the following day once compatible blood was secured. Intramuscular dexamethasone was given due to the risk of preterm delivery. Fetal sampling showed a hematocrit of 4.5%, and 25 mL of red cell concentrate was infused. The volume transfused was intentionally reduced from the calculated target to minimize the risk of circulatory overload.

The next day, Doppler assessment showed an MCA-PSV of 34.87 cm/s (1.13 MoM). A second IUT was performed three days later, with a pre-transfusion fetal hematocrit of 21%. A total of 22 mL of red blood cell concentrate was transfused.

**Figure 2:**
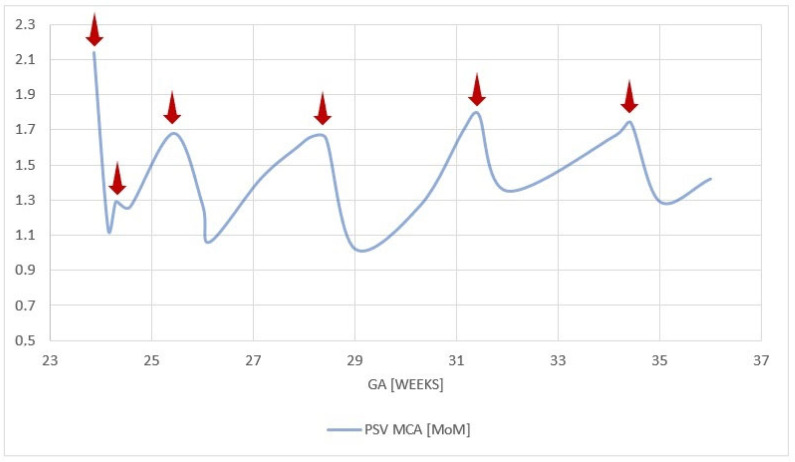
Longitudinal changes in fetal PSV MCA throughout pregnancy. Red arrows indicate the time points at which (IUTs were performed. PSV MCA—peak systolic velocity of the middle cerebral artery; GA—gestational age; IUT—intrauterine transfusion.

**Figure 3:**
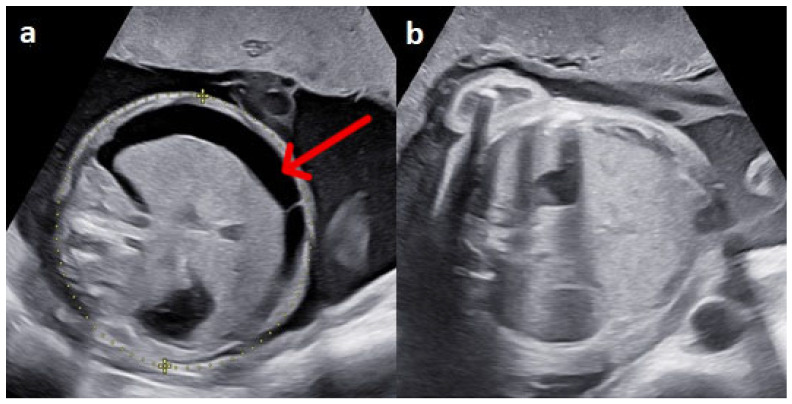
Resolution of fetal ascites after intrauterine transfusions (IUTs). Transverse ultrasound view of the fetal abdomen. (**a**) Massive fetal ascites (red arrow) at 24 weeks of gestation, at the time of hemolytic disease of the fetus diagnosis. (**b**) Complete resolution of ascites at 27 weeks of gestation following three IUTs.

The patient underwent four additional IUTs, with the final procedure performed at 34 + 6 weeks. Pre- and post-transfusion MCA-PSV values, pre-transfusion hematocrits, and transfused volumes are summarized in Table 1. Fetal condition was followed using serial MCA Dopplers (Figure 2) and cardiotocography. Sonographic findings progressively improved, with complete resolution of ascites and subcutaneous edema by 27 weeks (Figure 3).

#### 3.1.3. Outcomes

A cesarean section was performed at 36 weeks due to a contraindication to labor induction following two prior cesarean deliveries. A female neonate was born weighing 3190 g with Apgar scores of 10 at 1 and 5 min. Cord blood gases were normal. Phototherapy was initiated for hyperbilirubinemia (peak 7.50 mg/dL; term reference < 5 mg/dL) and continued for five days. No transfusion or NICU admission was required. Mother and infant were discharged in good condition on postpartum day 7.

## 4. Discussion

We report the case of a 35-year-old gravida 4, para 3 woman, whose first two pregnancies ended in uncomplicated term deliveries. HDFN was first recognized during her third pregnancy, occurring late in gestation and with a mild course that did not necessitate intrauterine transfusion (IUT). Her fourth pregnancy, however, was complicated by early-onset, severe HDFN with profound fetal anemia, markedly elevated MCA-PSV, and advanced hydrops. Despite an initially poor prognosis, the fetus demonstrated a robust response to serial IUTs, resulting in a favorable neonatal outcome.

The cause of maternal sensitization in the patient’s third pregnancy—the first affected by HDFN—cannot be definitively established. Possible explanations include omission of postpartum anti-D prophylaxis after the second delivery, inadequate prophylaxis during the third pregnancy, or an unrecognized fetomaternal hemorrhage exceeding the protective capacity of a standard 300-µg dose of anti-D immunoglobulin.

Sensitizing events can occur at any stage of pregnancy, including early pregnancy loss, ectopic pregnancy, first-trimester bleeding, or invasive procedures such as chorionic villus sampling or amniocentesis [17,18,19,20,21]. The risk of fetomaternal hemorrhage increases with gestational age, peaking in the third trimester and at delivery. Obstetric factors such as cesarean delivery, multifetal gestation, placenta previa, placenta accreta spectrum, or placental abruption further elevate this risk [22,23,24,25]. Although routine anti-D administration prevents most cases of HDFN, large or occult hemorrhages may compromise its efficacy [26,27,28]. Targeted testing—such as the rosette test, Kleihauer–Betke test, or flow cytometry—can identify women with substantial hemorrhage who may benefit from dose adjustment [29,30,31].

In the present case, no sensitizing events were documented, and the absence of prior obstetric records prevents confirmation of prophylaxis or occult hemorrhage. Therefore, all explanations for the initial maternal alloimmunization remain speculative.

Once HDFN is diagnosed, timely intrauterine transfusion (IUT) is essential and offers the highest survival rate [16,32]. Large cohort data from Zwiers et al. show substantial improvement in safety, with procedure-related perinatal mortality falling from 13% to 3% over time [33]. Timing must be individualized, particularly in severe hydrops, where delaying intervention may increase the risk of intrauterine demise.

An important element of managing HDFN is determining the timing of cordocentesis and intrauterine transfusion (IUT). MCA-PSV is a reliable non-invasive marker of fetal anemia, though its accuracy may decline after multiple transfusions or late in gestation [34,35]. In this case, MCA-PSV correlated well with fetal hematologic status, and the timing of each IUT was guided by Doppler values and the interval since the prior procedure. Retrospectively, all transfusions appeared appropriately timed, balancing procedural risk with the risk of fetal deterioration.

Timing of delivery is equally important in severe HDFN. Current evidence supports the safety of IUTs after 34 weeks, permitting pregnancy extension and reducing prematurity-related risks [36,37,38]. In this case, an uneventful IUT at 34 + 6 weeks and delivery at 36 weeks resulted in a favorable neonatal outcome, reinforcing the need for individualized late-gestation management.

Patients with a history of HDFN should receive contraceptive counseling to prevent complications in future pregnancies, as each subsequent gestation may trigger a stronger immune response and increase the risk of severe outcomes, including fetal demise. Documenting previous HDFN enables early fetal surveillance with MCA-PSV and timely planning of invasive procedures when indicated.

Overall, strategies for monitoring and managing HDFN require further investigation, as the limited number of cases hinders robust outcome analyses. The main limitation of our study is that it is a single case report, which precludes generalization of the findings to a larger population. Nevertheless, its strength lies in the comprehensive and detailed description of both the IUT methodology and the monitoring of a pregnancy complicated by HDFN, providing valuable insights for clinicians managing similar high-risk cases.

## 5. Conclusions

1. Initiating treatment in severely anemic, hydrops-affected fetuses can yield favorable outcomes.
2. MCA-PSV Doppler remains a reliable method to monitor fetal anemia, even after multiple transfusions, guiding clinical decision-making.
3. Late IUTs (after 34 weeks) can safely prolong pregnancy, mitigate prematurity-related complications, and improve neonatal outcomes.
4. Preventive measures, including effective anti-D prophylaxis and screening for significant fetomaternal hemorrhage with dose adjustment, are essential to reduce the risk of severe HDFN.

## Figures and Tables

**Table 1:** Detailed characteristics of each intrauterine transfusion (IUT), including gestational age (GA), fetal hematocrit (HCT), transfused blood volume, and PSV MCA (cm/s and MoM) before and after the procedure.

IUT No.	GA [Week + Day]	PSV MCA Day Before IUT [cm/s]	PSV MCA Day Before IUT [MoM]	Fetal HCT at the Day of IUT [%]	Volume of Blood Transfused [mL]	PSV MCA Day After IUT [cm/s]	PSV MCA Day After IUT [MoM]
1	24 + 0	65.31	2.14	4.5	25	34.87	1.13
2	24 + 2	40.19	1.29	22	22	39.60	1.26
3	25 + 5	55.00	1.68	20	35	42.85	1.27
4	28 + 6	62.06	1.65	21	46	39.30	1.02
5	31 + 5	77.61	1.79	23	60	59.98	1.35
6	34 + 6	86.58	1.74	21	90	65.90	1.29

## Data Availability

The original contributions presented in the study are included in the article, further inquiries can be directed to the corresponding authors.

## Abbreviations

The following abbreviations are used in this manuscript:

HDFN	Hemolytic disease of the fetus and newborn
IUT	Intrauterine transfusion
PSV MCA	Fetal middle cerebral artery peak systolic velocity
NICU	Neonatal intensive care unit
GA	Gestational age
